# Efficacy and Safety of Zuojin Pill for the Treatment of Chronic Nonatrophic Gastritis: A Randomized Active-Controlled Clinical Trial

**DOI:** 10.1155/2022/2266023

**Published:** 2022-04-07

**Authors:** Ruilin Wang, Yanling Wang, Zheng Lu, Jing Jing, Zhongxia Wang, Tingting He, Miao Tian, Zongyang Yuan, Yanfei Cui, Wenya Rong, Xiao Ma, Yanling Zhao

**Affiliations:** ^1^Division of Integrative Medicine, The Fifth Medical Center, General Hospital of PLA, Beijing 100039, China; ^2^Senior Department of Hepatology, The Fifth Medical Center, General Hospital of PLA, Beijing 100039, China; ^3^School of Chinese Medicine, Southern Medical University, Guangzhou 510515, China; ^4^School of Pharmacy, Chengdu University of Traditional Chinese Medicine, Chengdu 611137, China; ^5^Department of Pharmacy, General Hospital of PLA, Beijing 100039, China

## Abstract

**Objective:**

Zuojin pill (ZJP) is used as the classical prescription for a wide variety of digestive diseases. However, there is a lack of direct evidence for its use in the treatment of chronic nonatrophic gastritis (CNG). In particular, there is a lack of rigorous trials of randomized controlled designs. In this study, a randomized active-controlled clinical trial was performed to verify the efficacy and safety of ZJP in detail.

**Methods:**

Patients with CNG were divided into the ZJP group and the Marzulene-S granule group. Patients were enrolled from September 2019 to February 2021 (ChiCTR2000040549). Endoscopy and histology scores were evaluated as the primary outcome measure. The *Helicobacter pylori* positive rate and the disappearance rate of symptoms were also measured to reflect the outcomes. Finally, adverse events were also calculated as the index of safety.

**Results:**

A total of 68 eligible patients were enrolled in this trial and randomly divided into two groups with baseline comparability. ZJP was able to improve the red plaques as well as bile reflux scores compared with Marzulene-S granule (*P*=0.043 and *P*=0.019, respectively). Moreover, it also remarkably alleviated the active chronic inflammation score (*P*=0.043). However, there was no difference between the *Helicobacter pylori* positivity rate (*P*=0.752). The symptom scores of abdominal distension (*P*=0.004), belching (*P*=0.010), and loss of appetite (*P*=0.019) were alleviated by ZJP, but nausea and vomiting were not (*P*=0.616). ZJP can also be considered safe with no obvious adverse effects.

**Conclusion:**

ZJP might decrease mucosal injury and alleviate symptoms in CNG. In addition, more large-scale clinical trials should be carried out to further confirm its clinical efficacy and safety.

## 1. Introduction

Chronic gastritis refers to the chronic infiltration of inflammation in the gastric mucosa and is the most common disease worldwide. It can be divided into three types: nonatrophic gastritis (CNG), atrophic gastritis (CG), and other special types. Recent data from the *Digestive Endoscopy Branch of the Chinese Medical Association* indicated that approximately 49.3% of patients with upper gastrointestinal symptoms were diagnosed with CNG. Further evidence suggested that CNG can develop into CG without proper treatment. Patients who had CG with premalignant gastric lesions were shown to carry a remarkable risk of gastric cancer within 10 years of follow-up. Even in the short term, symptomatic patients might have a decreased quality of life due to nonspecific dyspeptic symptoms, such as epigastric discomfort, belching, distention, nausea, and even loss of appetite. However, there is still a lack of a specific medicine for CNG treatment due to the complex pathogenesis. *Helicobacter pylori* (*Hp*) infection is believed to be the primary cause of CNG, and therapy involving the eradication of *Hp* is applied worldwide. Clarithromycin-containing regimens are recommended as first-line drugs. However, the increasing rate of antibiotic resistance has already influenced the treatment of CNG [1]. Moreover, proton pump inhibitors (PPIs), such as omeprazole, esomeprazole, and lansoprazole, are also important medicines that could reduce the damage to the mucosa that is induced by gastric acid. Studies have indicated that PPIs are associated with a series of adverse effects, including osteoporosis, hypomagnesemia, *Clostridium difficile* colitis, and cardiovascular morbidity [2, 3]. In addition, bismuth quadruple therapy is also widely employed as a first-line treatment. However, current studies have suggested that it might cause short-term dysbiosis of the gut microbiota and lead to adverse effects [4]. Specific medicine with ideal efficacy that can improve the quality of life is urgently needed for CNG patients.

It has long been recognized that traditional medicine still demonstrates unique therapeutic characteristics in the modern clinical system [5, 6]. Artemisinin, derived from *Artemisia annua* L. (Qinghao), is one of the most commonly used medicines to efficiently fight against malaria. The original plant was first recorded by Ge Hong in *A Handbook of Prescriptions for Emergencies* and was applied to relieve malaria symptoms. It is widely thought to be a gift from traditional Chinese medicine to the world [7]. Apart from artemisinin, Maxingshigan decoction and Yinqiao powder have also been recently used to fight against complex diseases. These two prescriptions are thought to be antiplague formulae in traditional Chinese medicine. In 2011, the combination of these two formulae was demonstrated to reduce the fever resolution period of patients with H1N1 influenza virus infection compared with oseltamivir in a prospective randomized controlled trial [8]. Lianhuaqingwen capsule, one type of traditional Chinese patent medicine that is made from the combination of Maxingshigan decoction and Yinqiao powder, was further confirmed to be effective in alleviating coronavirus disease 2019 (COVID-19) pneumonia in the clinic [9]. Therefore, it could serve as a crucial source for drug development from traditional Chinese medicine.

Zuojin pill (ZJP) was initially created by Danxi Zhu in his *Danxi's Experiential Therapy,* which is a well-known formula that has been used since the 15th century. Two kinds of herbal medicine containing the root of *Coptischinensis* Franch. and the fruit of *Euodia ruticarpa* (A. Juss.) Benth. are involved in this prescription at a ratio of 6 : 1 (w/w). It is used for the pattern of liver fire invading the stomach, which represents specific symptoms, including heartburn, dyspepsia, vomiting, and abdominal distension, in traditional Chinese medicine. This pattern occurs in a wide variety of digestive diseases in modern clinics [10]. Hence, it is recognized as the principle medicine for the treatment of patients suffering from gastritis, gastroesophageal reflux disease, gastric ulcer, and even gastric carcinoma [11]. Several preclinical pharmacologic experiments also suggested that the Zuojin pill could significantly reduce gastric mucosal injury by inhibiting the regulation of the NF-кB signaling pathway and reducing the inflammatory response [12, 13]. All these studies provide indirect data that confirm the therapeutic efficacy of the Zuojin pill on CNG. However, there is a lack of direct evidence, especially with rigorous trials of randomized controlled designs. In this study, an open-label, randomized controlled trial was performed to verify the efficacy and safety of the Zuojin pill in detail. The results will definitely guide clinical applications.

## 2. Methods and Design

### 2.1. Participant Diagnosis

Patients with CNG were recruited from September 2019 to February 2021 at the Fifth Medical Center of PLA General Hospital, Beijing, China. The protocol was approved by the Ethics Committee at Fifth Medical Center of PLA General Hospital (approval number: 2019087D). All procedures and the purpose of the trial were clearly explained to all participants. Consent was obtained prior to participation. Participants with CNG were diagnosed according to the current standards of the Chinese Consensus on Chronic Gastritis (Shanghai, 2017).

#### 2.1.1. Clinical Manifestations

Nonspecific dyspepsia, such as epigastric pain, distention, belching, acid regurgitation, nausea, vomiting, and loss of appetite, was the clinical manifestations of symptomatic patients. The physical sign was slight epigastric pain or mild discomfort in the upper abdomen.

#### 2.1.2. Endoscopic and Histopathological Diagnosis

Endoscopic and histopathological examinations were the main references for CNG diagnosis. The essential characteristics of CNG are red plaques with punctuates, patches, and striae. Coarse and uneven mucosa, hemorrhagic spots or plaques, oedematous mucosa, and exudates without atrophic changes were also seen under endoscopy. Furthermore, no atrophic changes or intestinal metaplasia were found in the histopathological examination of the gastric mucosa.

### 2.2. Inclusion, Exclusion, Rejection, and Withdrawal Criteria

#### 2.2.1. Inclusion Criteria

The inclusion criteria were as follows: participants were diagnosed with CNG according to the Chinese Consensus on Chronic Gastritis; participants were diagnosed with liver fire invading stomach syndrome, according to TCM syndrome differentiation; participants were between 20 and 70 years of age; participants voluntarily provided written informed consent.

#### 2.2.2. Exclusion Criteria

The exclusion criteria were as follows: patients who underwent gastric-related surgery; patients with atrophic changes, intestinal metaplasia, or suspected malignant changes in the gastric mucosa; patients who also had other systemic diseases in the heart, liver, lung, kidney, or blood system; female patients who are planning to have a baby, are currently pregnant, or are lactating; patients with life-threatening illnesses, such as tumors and AIDS; patients who received other kinds of medicine in the 2 weeks before recruitment; and patients who were judged as inappropriate to be enrolled in the trial by investigators.

#### 2.2.3. Rejection Criteria

The rejection criteria were as follows: patients who were misdiagnosed; patients who did not take the proper drug dosage; and patients who could not complete the follow-up.

#### 2.2.4. Withdrawal Criteria

The withdrawal criteria were as follows: patients who quit the clinical trial voluntarily and patients who were intolerant to the Zuojin pill or conventional therapy. Adverse events occurred during the trial. These adverse events included severe hepatic injury, malignant tumors, or hemorrhage of the digestive tract, and thus, a patient with these events was unlikely to complete the procedure.

### 2.3. Intervention

Participants assigned to the control group received Marzulene-S granule (2.01 g/day, three times a day, for 12 weeks). One granule of Marzulene-S weighed 0.67 g and contained 663.3 mg L-glutamine and 2.0 mg sodium gualenate. Participants in the treatment group received the Zuojin pill (6.0 g/day, twice a day, for 12 weeks). Zuojin pill was available from the Department of Pharmacy in the Fifth Medical Center of PLA General Hospital. A chemical analysis was performed to ensure the homogeneity of the prescription. During the treatment, a healthy lifestyle with a light diet, regular work and rest, and proper exercise were required, and smoking, alcohol consumption, and the consumption of spicy foods were prohibited. Patients with *Helicobacter pylori* also received clarithromycin-containing regimens for eradication. A follow-up was scheduled every 4 weeks following the treatment. Patients received conventional treatment if they did not obtain full recovery of CNG after the trial.

### 2.4. Outcome Measures

#### 2.4.1. Primary Outcome

The score of endoscopy evaluation, involving red plaques, erosion, hemorrhage, and bile reflux, was compared between the control group and the treatment group (Supplementary Table 1). The grade of histopathological changes, including chronic inflammation and active chronic inflammation, was also evaluated (Supplementary Table 2). The standard was set according to the Chinese Consensus on Chronic Gastritis.

#### 2.4.2. Secondary Outcomes

*(1) Helicobacter pylori positive rate*. The *Helicobacter pylori* positive rate in patients was also evaluated for the treatment.

*(2) Symptom scores*. Changes in each symptom score, including abdominal distension, belching, nausea, vomiting, and loss of appetite, were compared to reflect the quality of life (Supplementary Table 3).

#### 2.4.3. Subgroup Analysis

Subgroup analysis based on *Hp* infection was also conducted with all the outcome measures.

### 2.5. Safety Outcome

Patients were interviewed every 4 weeks to record adherence and identify adverse events. Routine tests, including blood, urine, and stool tests, electrocardiogram, and liver and renal function tests, were performed at baseline. If the adverse events were relevant to the medication, patients were followed until their adverse events were resolved or there was no clinical significance.

### 2.6. Preparation and HPLC-MS/MS Analysis of Zuojin Pill

The preparation and HPLC-MS/MS analysis of the Zuojin pill were conducted according to a previous method.

#### 2.6.1. Preparation of Zuojin Pill Extracts and Standard Samples

The root of *Coptis chinensis* Franch. and the fruit of *Euodia ruticarpa* (A. Juss.) Benth. ley were weighed and added at 6 : 1 ratio. They were soaked for 30 min in pure water (1/10, w/v). After that, it was extracted twice by heating (1 h at a time). The filtrate was rotationally evaporated, condensed, and dried into a dry powder for preservation. The weight ratio of the Zuojin pill was 23.48%. Berberine hydrochloride, *Coptis chinensis* alkaloid, palmatine, magnolia alkaloid, evodiamine, rutaecarpine, and dehydroevodiamine were purchased from Chengdu Chroma-Biotechnology Co., Ltd. (China). Carbamazepine (internal standard) was purchased from the National Institutes for Food and Drug Control. Berberine hydrochloride, *Coptis chinensis* alkaloid, palmatine, magnolia alkaloid, evodiamine, rutaecarpine, and dehydroevodiamine were prepared to standard concentrations of 1.1, 5.15, 1.6, 2.55, 12, 17, and 0.5 *μ*g/mL in methanol/water (1 : 1, v : v). Aliquots of all standards and internal standards were stored at −20°C. Linearity was measured using a freshly prepared calibration pool of the mixed standards that was diluted to 1/2, 1/4, 1/10, 1/20, 1/40, 1/100, 1/500, and 1/1000 to obtain 9 final standard concentrations. The final concentration of the internal standard was 10 *μ*g/mL. Each concentration was prepared in three replicates. The calibration curve was prepared by determining the best fit of the peak area ratio (peak area of analyte/internal standard) versus concentration. The calibration curves were constructed using linear regression with 1/*x* or 1/*x*2 weighting factors. The results were used to calculate the overall linearity.

#### 2.6.2. HPLC-MS/MS Analysis

The chromatographic separation was carried out on a Shim-pack GIS C18 column (10 mm × 2.1 mm, 3 *μ*m) fitted on a Jasper HPLC system. The mobile phases were 0.1% formic acid (A) and acetonitrile (B). The flow rate was set to 0.3 mL/min. The column temperature was maintained at 40°C. The following gradient program was used: the initial mixture of 90% A and 10% B was held for 0.5 min; the linear gradient was increased to 70% B in 5 min and held for 2 min; then the linear gradient was increased to 90% B in 0.1 min and held for 1 min; then return to the initial conditions in 0.1 min, followed by 3.3 min of equilibration. The total run time was 12 min, and the injection volume was 2 *μ*L. Mass spectra were obtained using an API 3200 equipped with a TurboIon electrospray (ESI) interface set in the positive mode (needle voltage +5500 V). The mass spectrometry conditions were as follows: CUR, 20 psi; Gas 1 and Gas 2, 55 psi; IS: 5500 V; gas temperature: 550°C. The acquisition dwell time for each transition monitored was 50 ms.

### 2.7. Statistical Analyses

A series of statistical analyses were conducted by SPSS 26.0. Continuous variables are expressed as ‘mean ± standard', and categorical variables are expressed as frequencies (percentages). Student's *t*-test, Mann–Whitney test, or Chi-square test was used to compare baselines in different circumstances. For the comparison of variations from baseline to endpoint, Student's *t*-test and Wilcoxon signed-ranks test were performed according to normal or nonnormal variables, respectively. Moreover, the Wilcoxon test was applied to analyze the differences in the endoscopic scores and symptom scores between groups. The Chi-square test or Fisher's exact test was used for the symptom disappearance rate. All statistical tests were two-sided. The levels of *P* < 0.05 and *P* < 0.01 were assumed to be statistically significant and markedly significant, respectively.

## 3. Results

### 3.1. Participant Distribution

**Figure 1:**
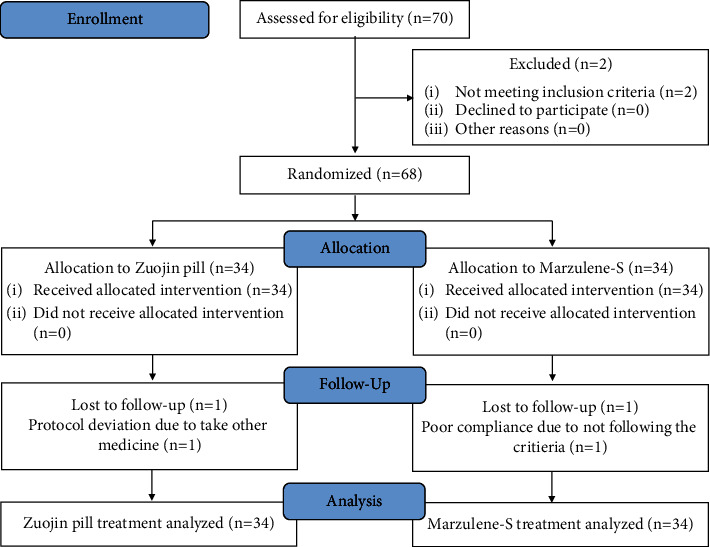
Standard CONSORT flowchart of the clinical trial.

Seventy patients with endoscopy evaluation were determined to be eligible. However, one patient had liver cirrhosis, and one patient refused to sign the informed consent forms. Thus, a total of 68 patients were eligible for inclusion in this trial according to the CONSORT statement (Figure 1). All 68 patients were randomly assigned to the control group and treatment group. Thirty-four patients were assigned to the treatment group. This group included 22 men and 12 women, and the average age was 56.20 ± 9.33 years. Thirty-four patients were included in the control group. This group included 17 men and 17 women, and the average age was 53.91 ± 10.92 years. Two patients were excluded due to loss of follow-up, 1 patient deviated from the protocol, and 1 patient had poor compliance.

### 3.2. Baseline Characteristics

There was no significant difference in age, sex, endoscopic scores, histopathological changes, or symptom scores between the two groups. Moreover, the number of patients with *H. pylori* (positive) in the treatment group (44.12%, 15/34) and that in the control group (52.94%, 18/34) were also comparable (*P*=0.355) (Table 1).

### 3.3. Endoscopy and Histology Evaluation Scores

Zuojin pill was able to improve the red plaques and the bile reflux scores compared with Marzulene-S granule (*P*=0.043 and *P*=0.019, respectively). However, there was no significant difference in the erosion score between patients who received the Zuojin pill and the Marzulene-S granule (*P*=0.769). Although the Zuojin pill had a trend of decreasing hemorrhage compared with Marzulene-S granule, it did not reach statistical significance (*P*=0.405) (Table 2). In addition, the grade of histopathological changes in chronic inflammation was not significantly different between the two therapies (*P*=0.055). However, the Zuojin pill markedly alleviated the active chronic inflammation score compared with the Marzulene-S granule (*P*=0.043) (Table 2).

### 3.4. *Helicobacter pylori* Positive Rate

**Figure 2:**
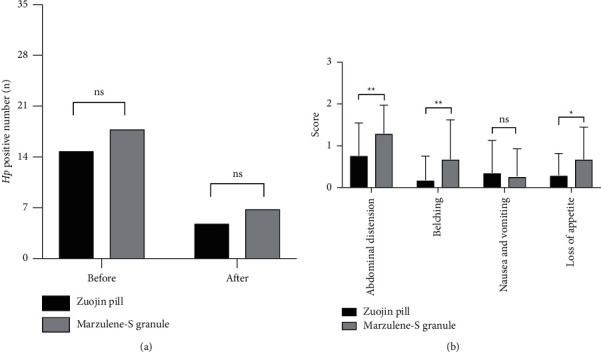
Comparison of secondary outcome measures. (a) *Helicobacter pylori* positive patients before and after treatment. (b) Symptom scores after treatment. ^*∗*^*P* < 0.05 compared with the Marzulene-S granule group; ^*∗∗*^*P* < 0.01 compared with the Marzulene-S granule group; ns: No significance).

There was no difference in the *Helicobacter pylori* positive rate between the patients in the Zuojin pill group (14.70%, 5/34) and those in the Marzulene-S granule group (20.59%, 7/34) (Figure 2(a)).

### 3.5. Symptom Scores

The disappearance rates of symptoms in patients treated with the Zuojin pill were 0.76 ± 0.78 for abdominal distension, 0.18 ± 0.58 for belching,0.35 ± 0.77 for nausea and vomiting, and 0.29 ± 0.52 for loss of appetite. These rates in the Marzulene-S granule group were 1.29 ± 0.68, 0.68 ± 0.94, 0.26 ± 0.66, and 0.68 ± 0.77, respectively. Zuojin pill significantly alleviated the symptoms of abdominal distension, belching, and loss of appetite compared with Marzulene-S granule (*P*=0.004, *P*=0.010, *P*=0.019). However, there was no difference in the symptoms of nausea and vomiting between the two groups (*P*=0.616) (Figure 2(b)).

### 3.6. Subgroup Analysis

Because *Hp* infection is thought to be the main cause of CNG, a subgroup analysis based on *Hp* infection was also conducted. The results indicated that the Zuojin pill could significantly decrease active chronic inflammation compared with Marzulene-S granule treatment in patients with *Hp* infection (*P*=0.033). Moreover, the Zuojin pill was able to alleviate red plaques (*P*=0.016), chronic inflammation (*P*=0.005) , and abdominal distension (*P*=0.012) compared with Marzulene-S granule treatment in patients without *Hp* infection (Table 3).

### 3.7. Adverse Events

Adverse events included constipation (1 case in the Zuojin pill group) and dizziness (1 case in the Zuojin pill group and 2 cases in the Marzulene-S granule group). None of the cases were determined to be related to the medication.

### 3.8. HPLC-MS Analysis of Zuojin Pill

Seven major compounds were identified, including berberine hydrochloride *Coptis chinensis* alkaloid, palmatine, magnolia alkaloid, evodiamine, rutaecarpine, dehydroevodiamine, and carbamazepine, which was the internal standard (Supplementary Figure 1).

## 4. Discussion

### 4.1. The Insight into CNG from Mechanism to Treatment

CNG is a common digestive disease but has a wide variety of complex mechanisms that require further investigation. The traditional concept of ‘one target, one disease' seems inadequate for the determination of this crucial pathogenesis and is a limited approach for treatment [14, 15]. Therefore, the recent recognition of the pathogenesis of this disease could be beneficial to improving the therapeutic options. It is widely believed that the progression of CNG, CAG, gastric intestinal metaplasia (GIM), and gastric cancer (GC) occurs through a thoroughly programmed deterioration with organized steps. CNG is accompanied by various biological factors, such as *Helicobacter pylori* infection, excessive inflammation, oxidative stress, and DNA damage and repair.

*Helicobacter pylori* is a Gram-negative pathogenic bacterium that has been found to colonize in the gastric mucosa of a large population worldwide. The progress of *Helicobacter pylori* infection is believed to be the initiating cause of gastritis, and this has been established for almost a century [16]. However, the investigation into its role in inducing inflammatory responses in gastritis has never ended. Traditional concepts recognize that *Helicobacter pylori* activates inflammation through various pathways that are associated with modifying TLR ligands [17]. Moreover, the most recent research indicates that *Helicobacter pylori* could exacerbate gastric inflammation by triggering C-type lectin receptors to modify host cholesterol [18]. *Helicobacter pylori* infection is widely associated with inflammation and the dysregulation of metabolites, which affects the progression of gastritis to a great extent. Oxidative stress, which plays a double-edged role, has also received increasing attention for its role in the progression of gastritis. First, redox homeostasis and antioxidant defense are highly needed to maintain homeostasis of the gastric epithelium due to excessive exposure to harsh conditions involving digestive enzymes, an aggressive pH environment, and bacteria [19]. For instance, the products of lipid peroxidation, including HNE and its protein/histidine adducts, are thought to be closely related to the pathogenesis of numerous gastric diseases [20]. Interestingly, a beneficial role has emerged for HNE in eradicating *Helicobacter pylori* infection. A study from Grasberger indicated that the dual oxidases (DUOX) enzyme complex was able to prevent gastric colonization by alleviating *Helicobacter pylori* and the inflammatory response [21]. In addition, DNA damage and repair are another important cause of chronic gastritis. The *p42.3* gene is a typical tumorigenesis promoter in various cancers [22]. It has been proven that both *Helicobacter pylori* and inflammatory factors can significantly enhance *p42.3* expression at the protein level. This gene was also positively associated with the severity of injury to the gastric mucosa. Thus, the damage caused by *p42.3* might be a significant regulator that affects CNG [23].

It is critically important to reverse the complex signaling network associated with CNG progression. The concept of a combination medication for CNG, such as triple therapy, is to maintain the surrounding stability. This concept has lasted at least 2000 years since the development of traditional Chinese medicine. Zuojin pill is used to treat various gastric diseases that are associated with network regulation. Its gastroprotective effect has been revealed at the molecular level during the past decade. Rhizoma Coptidis, one of the constituents in the Zuojin pill, was able to alleviate chronic gastritis with *Helicobacter pylori* infection. Our previous pharmacological research revealed that this effect was due to palmatine. It might ameliorate *Helicobacter pylori*-induced chronic gastritis by inhibiting MMP-10 through the ADAM17/EGFR signaling pathway [24]. Moreover, this action was also related to a metabolite network involving taurine and hypotaurine metabolism, glycerophospholipid metabolism, and pentose and glucuronate interconversions [25]. Another compound, berberine, which is derived from Rhizoma Coptidis, also demonstrates a beneficial effect on chronic gastritis. Research has indicated that berberine is able to suppress the IRF8-IFN-*γ* signaling axis to inhibit progression [26]. Apart from Rhizoma Coptidis, the research on the effects of Fructus Evodiae on gastritis has also vastly improved. Rutaecarpine, one of the main quinazolino carboline alkaloids, exerts a remarkable gastroprotective effect against mucosal injury induced by ethanol. This effect is highly associated with the suppression of the nuclear translocation of NF-*κ*B p65 and its downstream signaling factors. The antioxidant role of this compound also involves the activation of the PI3K/AKT signaling pathway [27]. Thus, the components of the Zuojin pill are integral to the mechanisms of its effects.

### 4.2. The Guidance of TCM from Bedside to Bench to Bedside

Establishing an approach that connects basic research and clinical practice has long been an aim in medical science. In the past two decades, translational medicine has been considered a suitable concept for bridging this gap. It acts as a robust bidirectional link between research and application to enhance the rapid clinical translation and provide further feedback for basic research results [28]. During this progress, basic research knowledge can be translated to clinical application. It thus provides more advanced concepts, techniques, tools, and methods to address the diagnosis and treatment of diseases. Moreover, clinical researchers also ruminate the basic advances to further amend deficiencies in basic research. These relationships are commonly called ‘from bench to bedside', ‘from bedside to bench', or ‘B2B' [29]. Thereafter, chemicals were developed for various diseases via this approach [30].

As the cornerstone of traditional medicine, herbal medicine has been recognized as an important source for drug development [31, 32]. Moreover, modern basic research offers deep insight into this clinical application and promotes further indication of its use. Thus, many medicines experience the process of ‘from bedside to bench to bedside' to find the optimal guidance in TCM. The anti-influenza virus efficacy of Yinqiao powder (Yinqiaosan) is an outstanding example. This formula is found in ‘*Wen Bing TiaoBian*', which is the basis of the theoretical research on the warm-heat disease in TCM. It consists of nine herbal components, such as *Flos Lonicerae*, *Fructus forsythiae*, *Fructus arctii*, and *Herba Schizonepetae*. Yinqiao powder and its modified formulae have a long history in the treatment of the common cold, fever, coughing, and other respiratory infectious diseases. Furthermore, researchers found that Yinqiao powder is able to inhibit the influenza-A virus based on an infected embryonated hen egg model [33]. In 2011, a modified formula of Yinqiao powder demonstrated remarkable efficacy in reducing the time to fever resolution in patients combating H1N1 influenza virus infection in the clinic [8]. This model sheds light on the translational medicine approach for TCM and bridges the gap between clinical experience and medicinal therapy. In addition, our previous research also confirmed the gastroprotective effect of the Zuojin pill and its components against mucosal injury. Thus, a clinical trial will provide clinical evidence for the value of the Zuojin pill for further application in CNG.

### 4.3. The Indication of Current Research from Results to Application

This research mainly focused on the efficacy of the Zuojin pill on CNG in the clinic. The results suggested that the Zuojin pill was able to alleviate several indices of mucosal injury under endoscopy and histology. It could downregulate red plaques and bile reflux but not erosion and hemorrhage, indicating the selectivity of Zuojin pill action. In addition, it could remarkably alleviate active chronic inflammation. This action is critically important in anti-inflammation. Therefore, this efficacy coincides with our previous experimental results [27]. Furthermore, the efficacy of the Zuojin pill in alleviating symptoms of abdominal distension, belching, and loss of appetite was remarkable, indicating an integral improvement in quality of life. However, this result should be interpreted with caution. The concept of ‘pattern' is the core factor for differentiating disease and further treatment in traditional Chinese medicine. That is, the Zuojin pill might be more suitable for CNG patients with a pattern of liver fire invading the stomach in TCM. Patients with this kind of pattern might achieve higher efficacy. Thus, subgroup trials with different patterns are needed for further exploration. Regarding the *Helicobacter pylori* positivity rate, the Zuojin pill did not demonstrate a significant difference compared to the Marzulene-S granule. This is because the main targets of the Zuojin pill focus more on downstream signaling pathways involved in inflammatory regulation. Furthermore, safety was also observed during the whole trial. However, all the cases with adverse events were ultimately determined to have no relation to the medication. Therefore, Zuojin pill treatment could be considered safe.

### 4.4. The Limitations of the Current Research

Overall, this trial strictly abided by the clinical standard from design to the determination to calculation. However, there still exists improvement in three aspects. First, a multicenter randomized clinical trial with large sample size is needed for further exploration. Only by performing this kind of trial can the evidence reported here be applied to guide gastritis treatment. Second, this research mainly uses the modern clinical index of CNG. Zuojin pill alleviates various symptoms. Therefore, the index of TCM symptoms should also be evaluated to obtain further clinical data. Finally, a comprehensive investigation of the specific mechanisms is still needed. Due to the current progress of multiomics techniques, the exploration of combined metabolomics and proteomics is urgently needed to predict possible molecular mechanisms and indicate the cure index.

## 5. Conclusion

Zuojin pill is able to decrease the mucosal injury of CNG and alleviate the related symptoms to elevate the life of quality. It is also safe for patients to use. In addition, more large-scale clinical trials with longer intervention durations should be carried out to further prove its clinical efficacy and safety.

## Figures and Tables

**Table 1:** Comparison of baseline characteristics (mean ± SD).

Variables	Zuojin pill (*n* = 34)	Marzulene-S granule (*n* = 34)	*P* value
Gender (male/female)	22/12	17/17	0.327
Age	56.20 ± 9.33	53.91 ± 10.92	0.355
Red plaques	2.09 ± 0.83	2.15 ± 0.92	0.770
Erosion	2.09 ± 0.83	2.15 ± 0.66	0.747
Hemorrhage	1.62 ± 1.10	1.38 ± 0.95	0.350
Bile reflux	1.65 ± 0.69	1.76 ± 0.85	0.535
Chronic inflammation	2.35 ± 0.64	2.26 ± 0.66	0.581
Active chronic inflammation	2.20 ± 0.81	2.06 ± 0.78	0.447
Positive *Hp* rate	15 (44.1%)	18 (52.9%)	0.628
Abdominal distension	2.82 ± 0.39	2.68 ± 0.59	0.228
Belching	2.15 ± 0.36	2.29 ± 0.46	0.148
Nausea and vomiting	2.47 ± 0.67	2.38 ± 0.74	0.594
Loss of appetite	2.79 ± 0.50	2.76 ± 0.50	0.804

Notes: *Hp*, *Helicobacter pylori*.

**Table 2:** Comparison of endoscopy and histology evaluation after treatment (mean ± SD).

Variables	Zuojin pill (*n* = 34)	Marzulene-S granule (*n* = 34)	*P* value
Red plaques	1.15 ± 0.92	1.62 ± 0.95	0.043
Erosion	1.82 ± 0.83	1.88 ± 0.81	0.769
Hemorrhage	0.38 ± 0.74	0.53 ± 0.71	0.405
Bile reflux	0.26 ± 0.57	0.74 ± 0.99	0.019
Chronic inflammation	1.47 ± 0.96	1.91 ± 0.90	0.055
Active chronic inflammation	1.29 ± 1.12	1.82 ± 1.00	0.043

**Table 3:** Subgroup comparison based on *Helicobacter pylori* infection after treatment (mean ± SD).

Variables	Zuojin pill	Marzulene-S granule	*P* value
*Helicobacter pylori infection positive*	*N* = 15	*N* = 18	
Red plaques	1.40 ± 1.12	1.56 ± 0.92	0.665
Erosion	2.07 ± 0.80	2.11 ± 0.83	0.877
Hemorrhage	0.53 ± 0.83	0.67 ± 0.77	0.636
Bile reflux	0.47 ± 0.74	0.83 ± 1.04	0.263
Chronic inflammation	1.87 ± 0.99	1.78 ± 0.88	0.786
Active chronic inflammation	1.27 ± 1.10	2.06 ± 0.94	0.033
Abdominal distension	1.00 ± 0.84	1.39 ± 0.70	0.157
Belching	0.27 ± 0.70	0.78 ± 1.00	0.107
Nausea and vomiting	0.54 ± 0.92	0.39 ± 0.78	0.627
Loss of appetite	0.40 ± 0.63	0.83 ± 0.78	0.095

*Helicobacter pylori infection negative*	*N* = 19	*N* = 16	
Red plaques	0.95 ± 0.70	1.69 ± 1.01	0.016
Erosion	1.63 ± 1.01	1.62 ± 0.72	0.980
Hemorrhage	0.26 ± 0.65	0.38 ± 0.62	0.609
Bile reflux	0.16 ± 0.50	0.38 ± 0.88	0.369
Chronic inflammation	1.16 ± 0.83	2.06 ± 0.93	0.005
Active chronic inflammation	1.32 ± 1.16	1.56 ± 1.03	0.514
Abdominal distension	0.58 ± 0.69	1.19 ± 0.66	0.012
Belching	0.10 ± 0.46	0.56 ± 0.89	0.077
Nausea and vomiting	0.21 ± 0.63	0.12 ± 0.50	0.664
Loss of appetite	0.21 ± 0.42	0.50 ± 0.73	0.152

## Data Availability

Data are available in ChiCTR2000040549.

## Supplementary Materials

Supplementary Materials Supplementary Table 1: score of endoscopy evaluation. Supplementary Table 2: grade of histopathological changes. Supplementary Table 3: symptom scores evaluation. Supplementary Figure 1: the HPLC-MS analysis of the Zuojin pill.

## References

[1] Schubert J. P., Gehlert J., Rayner C. K. (2021). Antibiotic resistance of *Helicobacter pylori* in Australia and New Zealand: a systematic review and meta‐analysis. *Journal of Gastroenterology and Hepatology*.

[2] Liu Y., Zhu X., Li R., Zhang J., Zhang F. (2020). Proton pump inhibitor ugeniang and potentially inappropriate prescribing analysis: insights from a single-centred retrospective study. *BMJ Open*.

[3] Sattayalertyanyong O., Thitilertdecha P., Auesomwang C. (2020). The inappropriate use of proton pump inhibitors during admission and after discharge: a prospective cross-sectional study. *International Journal of Clinical Pharmacy*.

[4] Hsu P.-I., Pan C.-Y., Kao J. Y. (2018). *Helicobacter pylori* eradication with bismuth quadruple therapy leads to dysbiosis of gut microbiota with an increased relative abundance of Proteobacteria and decreased relative abundances of Bacteroidetes and Actinobacteria. *Helicobacter*.

[5] Law S., Leung A. W., Xu C. (2020). Is traditional Chinese medicine, “radix IlicisPubescentis” possible for treating cardiovascular disease?. *Traditional and Integrative Medicine*.

[6] Nayebi N., Esteghamati A., Meysamie A. (2019). The effects of a Melissa officinalis L. based product on metabolic parameters in patients with type 2 diabetes mellitus: a randomized double-blinded controlled clinical trial. *Journal of Complementary & Integrative Medicine*.

[7] Tu Y. (2016). Artemisinin-A gift from traditional Chinese medicine to the world (nobel lecture). *Angewandte Chemie International Edition*.

[8] Wang C., Cao B., Liu Q.-Q. (2011). Oseltamivir compared with the Chinese traditional therapy maxingshigan-yinqiaosan in the treatment of H1N1 influenza. *Annals of Internal Medicine*.

[9] Chen X., Wu Y., Chen C. (2021). Identifying potential anti-COVID-19 pharmacological components of traditional Chinese medicine Lianhuaqingwen capsule based on human exposure and ACE2 biochromatography screening. *Acta Pharmaceutica Sinica B*.

[10] Guo W., Huang J., Wang N. (2019). Integrating network pharmacology and pharmacological evaluation for deciphering the action mechanism of herbal formula Zuojin pill in suppressing hepatocellular carcinoma. *Frontiers in Pharmacology*.

[11] Li S., Huang M., Wu G. (2020). Efficacy of Chinese herbal formula sini Zuojin decoction in treating gastroesophageal reflux disease: clinical evidence and potential mechanisms. *Frontiers in Pharmacology*.

[12] Wang Q. S., Zhu X. N., Jiang H. L., Wang G. F., Cui Y. L. (2015). Protective effects of alginate-chitosan microspheres loaded with alkaloids from Coptis chinensis Franch. And Evodia rutaecarpa (Juss.) Benth. (Zuojin Pill) against ethanol-induced acute gastric mucosal injury in rats. *Drug Design, Development and Therapy*.

[13] Wang J., Zhang T., Zhu L., Ma C., Wang S. (2015). Anti-ulcerogenic effect of Zuojin Pill against ethanol-induced acute gastric lesion in animal models. *Journal of Ethnopharmacology*.

[14] Schadt E. E., Friend S. H., Shaywitz D. A. (2009). A network view of disease and compound screening. *Nature Reviews Drug Discovery*.

[15] Ma X., Jiang Y., Zhang W. (2020). Natural products for the prevention and treatment of cholestasis: a review. *Phytotherapy Research*.

[16] Fock K. M., Graham D. Y., Malfertheiner P. (2013). *Helicobacter pylori* research: historical insights and future directions. *Nature Reviews Gastroenterology & Hepatology*.

[17] Nagai S., Mimuro H., Yamada T. (2007). Role of Peyer’s patches in the induction of *Helicobacter pylori*-induced gastritis. *Proceedings of the National Academy of Sciences*.

[18] Nagata M., Toyonaga K., Ishikawa E. (2021). *Helicobacter pylori* metabolites exacerbate gastritis through C-type lectin receptors. *Journal of Experimental Medicine*.

[19] Kanner J., Lapidot T. (2001). The stomach as a bioreactor: dietary lipid peroxidation in the gastric fluid and the effects of plant-derived antioxidants. *Free Radical Biology and Medicine*.

[20] Cherkas A., Zarkovic N. (2018). 4-Hydroxynonenal in redox homeostasis of gastrointestinal mucosa: implications for the stomach in health and diseases. *Antioxidants*.

[21] Grasberger H., El–Zaatari M., Dang D. T., Merchant J. L. (2013). Dual oxidases control release of hydrogen peroxide by the gastric epithelium to prevent Helicobacter felis infection and inflammation in mice. *Gastroenterology*.

[22] Mao L., Sun W., Li W. (2014). Cell cycle-dependent expression of p42.3 promotes mitotic progression in malignant transformed cells. *Molecular Carcinogenesis*.

[23] Chen P., Cui Y., Fu Q. Y., Lu Y. Y., Fang J. Y., Chen X. Y. (2015). Positive relationship betweenp42.3gene and inflammation in chronic non-atrophic gastritis. *Journal of Digestive Diseases*.

[24] Chen X., Wang R., Bao C. (2020). Palmatine ameliorates Helicobacter pylori-induced chronic atrophic gastritis by inhibiting MMP-10 through ADAM17/EGFR. *European Journal of Pharmacology*.

[25] Chen X., Zhang J., Wang R. (2020). UPLC-Q-TOF/MS-Based serum and urine metabonomics study on the ameliorative effects of palmatine on Helicobacter pylori-induced chronic atrophic gastritis. *Frontiers in Pharmacology*.

[26] Yang T., Wang R., Zhang J. (2020). Mechanism of berberine in treating *Helicobacter pylori* induced chronic atrophic gastritis through IRF8-IFN-*γ* ugeniang axis suppressing. *Life Sciences*.

[27] Ren S., Wei Y., Wang R. (2020). Rutaecarpine ameliorates ethanol-induced gastric mucosal injury in mice by modulating genes related to inflammation, oxidative stress and apoptosis. *Frontiers in Pharmacology*.

[28] Chen F.-M., Zhao Y.-M., Jin Y., Shi S. (2012). Prospects for translational regenerative medicine. *Biotechnology Advances*.

[29] Moore D. R. (2008). Reverse translation: clearing a path from bedside to bench. *Nature*.

[30] Gabizon A., Shmeeda H., Tahover E. (2020). Development of promitil, a ugenia prodrug of mitomycin c in PEGylated liposomes: from bench to bedside. *Advanced Drug Delivery Reviews*.

[31] Mosavat S. H., Heydari M., Hashempur M. H., Dehghani S. M. (2018). Use of complementary and alternative medicine among paediatric patients with hepatogastrointestinal diseases. *Eastern Mediterranean Health Journal*.

[32] Parveen S., Khan A. A., Khan Q. A. (2021). Antihyperlipidemic effect of seeds of jamun (ugenia jambolana) in subjects of intermediate hyperglycemia: a pilot study. *Traditional and Integrative Medicine*.

[33] Wang X., Hao O., Wang W., Ying X., Wang H. (2010). Evaluation of the use of different solvents to extract the four main components of Yinqiaosan and their in vitro inhibitory effects on influenza-A virus. *The Kaohsiung Journal of Medical Sciences*.

