# Supplementary material for: Efficacy and Safety of Zuojin Pill for the Treatment of Chronic Nonatrophic Gastritis: A Randomized Active-Controlled Clinical Trial
Source: Evid Based Complement Alternat Med. 2022 Apr 7;2022:2266023. doi: 10.1155/2022/2266023 (PMC9010182; doi:10.1155/2022/2266023)
Supplement: Supplementary Materials — Supplementary Table 1: score of endoscopy evaluation. Supplementary Table 2: grade of histopathological changes. Supplementary Table 3: symptom scores evaluation. Supplementary Figure 1: the HPLC-MS analysis of the Zuojin pill. [file 2266023.f1.docx]

**Supplementary Table 1 Score of endoscopy evaluation**

| **Item** | **Grade** | **Score** |
| --- | --- | --- |
| Red plaques | No red plaques in gastric mucosa | 0 |
|  | Red plaques with sporadic or interrupted linear type in gastric mucosa | 1 |
|  | Intensive plaques or linear series type in gastric mucosa | 2 |
|  | Red plaques with extensive and obvious fusion type in gastric mucosa | 3 |
| Erosion | No ruptured erosion | 0 |
|  | Sporadic erosion in gastric mucosa | 1 |
|  | Local, multiple erosion in gastric mucosa and no more than five area | 2 |
|  | Extensive, multiple erosion in gastric mucosa and more than five area | 3 |
| Hemorrhage | No flattened hemorrhagic spots or plaques in gastric mucosa | 0 |
|  | Local hemorrhagic spots or plaques in gastric mucosa | 1 |
|  | Multiple sites of hemorrhagic spots or plaques in gastric mucosa | 2 |
|  | Widespread hemorrhagic spots or plaques in gastric mucosa | 3 |
| Bile reflux | No bile flow back into stomach | 0 |
|  | Mild yellow bile flow back into stomach or yellowish mucinous lake | 1 |
|  | Middle yellow bile flow back into stomach | 2 |
|  | Massive yellow bile flow back into stomach | 3 |

Notes: This table referred the standard in Chinese Consensus on Chronic Gastritis and the literature of ‘XX Zhang, WW Chen, B She, et al. The efficacy and safety of Jian-Wei-Qu-Tong Pills for the treatment of chronic non-atrophic gastritis (spleen and stomach qi deficiency with damp-heat stasis syndrome): study protocol for a phase II, randomized controlled trial. Trial. 2014, 15: 272’.

**Supplementary Table 2 Grade of histopathological changes**

| **Item** | **Grade** | **Score** |
| --- | --- | --- |
| Chronic inflammation | Normal mononuclear cells in gastric mucosa | 0 |
|  | No more than 1/3 of chronic inflammatory cells localized in the mucosa | 1 |
|  | More than 1/3 but no more than 2/3 of chronic inflammatory cells localized in the mucosa | 2 |
|  | Dense accumulation of chronic inflammatory cells in the whole mucosa | 3 |
| Active chronic inflammation | Normal neutrophils in mucosal layer | 0 |
|  | No more than 1/3 of neutrophils in lamina propria of the mucosa | 1 |
|  | More than 1/3 but no more than 2/3 of neutrophils in the mucosal layer and in superficial, pit as well as glandular epithelial cells. | 2 |
|  | Dense infiltration of neutrophils, abscess with moderate activity. | 3 |

Notes: This table referred the standard in Chinese Consensus on Chronic Gastritis and the literature of ‘XX Zhang, WW Chen, B She, et al. The efficacy and safety of Jian-Wei-Qu-Tong Pills for the treatment of chronic non-atrophic gastritis (spleen and stomach qi deficiency with damp-heat stasis syndrome): study protocol for a phase II, randomized controlled trial. Trial. 2014, 15: 272’.

**Supplementary Table 3 Symptom scores evaluation**

| **Item** | **Grade** | **Score** |
| --- | --- | --- |
| Abdominal distension | None | 0 |
|  | Mild but not affecting life | 1 |
|  | Moderate but not affecting life | 2 |
|  | Severe and affecting life, need drug control | 3 |
| Belching | None | 0 |
|  | Mild | 1 |
|  | Moderate | 2 |
|  | Severe and affecting life | 3 |
| Nausea and vomiting | None | 0 |
|  | Present, but not very intense | 1 |
|  | Tolerable but intense | 2 |
|  | Very intense and unbearable, need drug control | 3 |
| Loss of appetite | None | 0 |
|  | Food intake reduced by 1/3 | 1 |
|  | Food intake reduced by 1/3 to 2/3 | 2 |
|  | Food intake reduced by 2/3 | 3 |

Notes: This table referred the standard in Chinese Consensus on Chronic Gastritis.


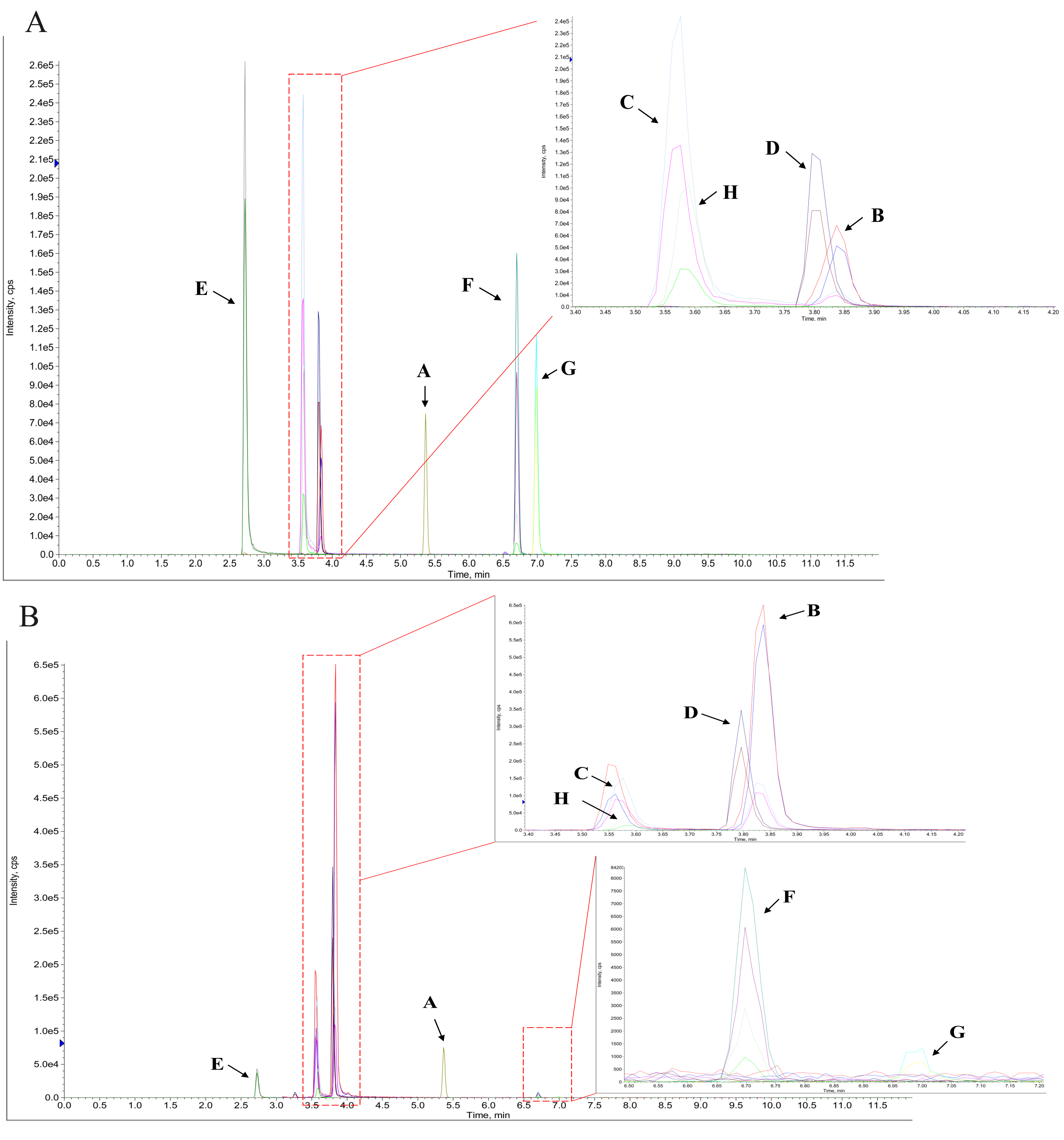


**Supplementary Figure 1 The HPLC-MS analysis of Zuojin pill**

The LC-MS of seven standard compounds (A) and Zuojin pill sample (B). Seven major compounds were identified. (a) Carbamazepine (internal standard). (b) Berberine hydrochloride. (c) Coptis chinensis alkaloid. (d) Palmatine. (e) Magnolia alkaloid. (f) Evodiamine. (g) Rutaecarpine. (h) Dehydroevodiamine
